# Heteroresistance: A cause of unexplained antibiotic treatment failure?

**DOI:** 10.1371/journal.ppat.1007726

**Published:** 2019-06-06

**Authors:** Victor I. Band, David S. Weiss

**Affiliations:** 1 Emory Antibiotic Resistance Center, Emory University, Atlanta, Georgia, United States of America; 2 Emory Vaccine Center, Emory University, Atlanta, Georgia, United States of America; 3 Division of Infectious Diseases, Department of Medicine, Emory University School of Medicine, Emory University, Atlanta, Georgia, United States of America; Duke University School of Medicine, UNITED STATES

Antibiotic resistance is a major and increasing healthcare problem that threatens many of the achievements of modern medicine. Transplants, chemotherapy, and survival of extremely premature infants all rely on the efficacy of antibiotics to fight off infection. According to the Centers for Disease Control and Prevention (CDC), over 2 million infections every year in the United States are caused by antibiotic-resistant bacteria, resulting in at least 23,000 deaths and US$55 billion in increased healthcare costs and lost productivity [1]. Without significant action, antibiotic-resistant infections could annually kill 10 million people worldwide by the year 2050, eclipsing the number of deaths caused by cancer and adding US$100 trillion to the world’s healthcare costs by that time [2]. It is therefore critical to understand novel and often underrecognized mechanisms of resistance that represent barriers to antibiotic efficacy so as to combat them with new drugs and therapeutic approaches.

## Heteroresistance as a form of subpopulation-mediated resistance

**Fig 1:**
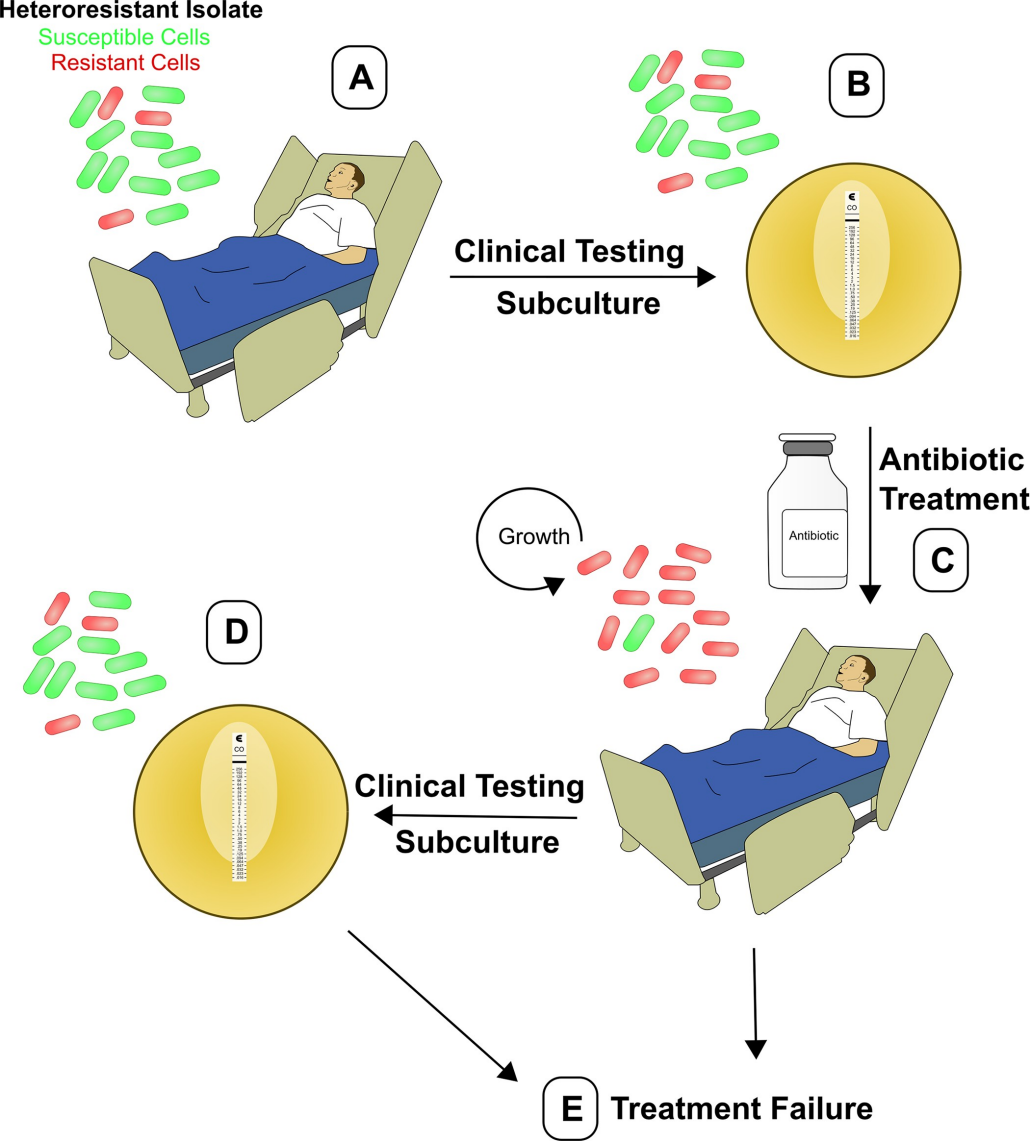
Dynamics of heteroresistance during infection and clinical susceptibility testing. (A) Schematic showing a patient infected with a heteroresistant bacterial isolate harboring a minor subpopulation of antibiotic-resistant cells (red). (B) Clinical susceptibility testing may not detect the minor resistant subpopulation, and the isolate would therefore be incorrectly designated susceptible to the given antibiotic. (C) Subsequent antibiotic therapy selects for the resistant subpopulation, which is able to grow in the presence of the drug. (D) Continued clinical susceptibility testing involves in vitro subculture of the heteroresistant bacteria in the absence of an antibiotic, which leads to a contraction of the resistant subpopulation. This minor subpopulation is therefore still not detected by clinical testing, and the isolate again appears susceptible. (E) Inappropriate antibiotic therapy ultimately leads to treatment failure and inability to clear the infection.

Studies on mechanisms of antibiotic resistance have typically focused on stable genetic mutations or acquisition of antibiotic resistance genes, both of which confer resistance to all the cells within a population. However, there is an increasing appreciation of the ways in which phenotypic traits expressed by minor subpopulations of cells can impact bacterial physiology, including resistance to antibiotics [3]. Heteroresistance (HR) is a phenomenon in which a preexisting subpopulation of resistant cells (Fig 1, panel A) can rapidly replicate in the presence of a given antibiotic (Fig 1, panel C), whereas the majority population of susceptible cells is killed. The mechanisms underlying HR are somewhat unclear, although unstable amplification of antibiotic resistance genes resulting in increased gene dosage is responsible for the resistant subpopulation in numerous cases [4–6].

HR is distinct from other forms of subpopulation-mediated resistance such as persistence, in which a small subpopulation of bacteria that are temporarily quiescent or very slow growing display increased resistance to a wide range of antibiotics [7]. Persistence is believed to lead to the relapse of infection after cessation of antibiotic therapy, but it is not capable of causing acute treatment failure. HR is also distinct from tolerance, wherein a whole population of bacteria are able to survive transient exposure to high antibiotic concentrations, and in which there may be no preexisting resistant cells prior to antibiotic exposure [7]. It should be noted that the term “heteroresistance” has been used to describe mixed populations of bacteria with stable genetic differences, including closely related bacteria that developed mutations [8] or coinfections with two unrelated strains [9]. These are distinct occurrences that are not the focus of this review.

## HR can affect treatment outcome

The relevance of HR has been debated; can the minor subpopulation of resistant cells affect treatment outcomes? There are numerous examples of vancomycin HR in *Staphylococcus aureus*, and it has been postulated that this may cause vancomycin treatment failure [10]. Although some reports suggest that vancomycin HR can lead to negative treatment outcomes [11], others have found that vancomycin is still effective in treating such strains [12]. These discrepancies may be due to the relatively minor 2- to 4-fold differences in minimum inhibitory concentration between the susceptible and resistant subpopulations in most vancomycin HR isolates. Beyond *S*. *aureus*, few studies have investigated the impact of HR on the outcome of in vivo antibiotic therapy.

We recently identified a clinical isolate of the nosocomial pathogen *Enterobacter cloacae* that exhibited HR to the last-line polymyxin antibiotic colistin [13]. Colistin has been used increasingly to treat infections caused by gram-negative bacteria that are resistant to carbapenems, including *Enterobacter*, *Klebsiella*, and *Escherichia coli*, which together constitute the majority of the carbapenem-resistant Enterobacteriaceae (CRE). Whereas most of the cells of the heteroresistant *E*. *cloacae* strain were highly susceptible to colistin, 1%–10% of the population was resistant to 1,000× higher concentrations [13]. These resistant cells rapidly replicated in the presence of colistin, quickly becoming the majority population, indicating that they are distinct from persisters [14]. Interestingly, the frequency of the resistant subpopulation returned to baseline after a single antibiotic-free subculture, highlighting that this subpopulation transiently expands in the presence of the drug. Although the basis for this phenotypic reversion was unclear, it may in some cases be due to the loss of the aforementioned multiplication of antibiotic resistance genes through an undefined mechanism. At the transcriptional level, the resistant and susceptible subpopulations exhibited vast differences, including the up-regulation of colistin resistance genes controlled by the two-component histidine kinase PhoQ in the resistant cells [13].

Most importantly, HR had a profound impact on the efficacy of antibiotic treatment in vivo. Whereas colistin was effective in rescuing mice infected with a susceptible strain, those infected with colistin HR isolates failed colistin therapy and were unable to survive [14]. Treatment failure was preceded by a significant expansion of the colistin-resistant subpopulation in vivo. These findings clearly demonstrate the profound effect that HR can have on antibiotic treatment outcomes.

## HR in the clinic

Although HR has been observed worldwide, in a variety of pathogens, and in response to numerous classes of antibiotics, very little epidemiologic data exists about its prevalence. In addition to *E*. *cloacae* and *S*. *aureus*, HR has been detected in *Klebsiella* species, *E*. *coli*, *Acinetobacter baumannii*, *Pseudomonas aeruginosa*, and others [15]. Furthermore, it has been observed against diverse antibiotics including aminoglycosides, carbapenems, and other beta-lactams [5, 15]. In a study of *E*. *coli* isolates in southwest China, 3.9% were heteroresistant to meropenem, 17.2% to ertapenem, and 25.0% to imipenem [16]. Additionally, *A*. *baumannii* strains from a cohort of Spanish hospitals displayed imipenem HR (20%) and meropenem HR (24%) [17].

HR has often been observed against the polymyxins colistin and polymyxin B. In addition to colistin HR in *E*. *cloacae* [14], this phenomenon has been described in many other species, including *A*. *baumannii* [18], *Klebsiella pneumoniae* [19], and *Stenotrophomonas maltophilia* [20], whereas polymyxin B HR has been observed in numerous species as well [15]. Polymyxin treatment in vitro can in some cases lead to regrowth of bacteria after initial killing and has been attributed to the presence of unstable resistant subpopulations characteristic of HR [14, 19, 21]. These subpopulations may be linked to the long history of polymyxin susceptibility testing being described as unreliable and difficult to interpret [22]. Clinical treatment with polymyxins is already challenging because of their low therapeutic index and drug stability [23], and the added complication of HR further threatens the clinical use of these last-line drugs.

There is a large gap in our understanding of the impact of HR on treatment efficacy in human patients, and it is thus imperative that future studies investigate its clinical relevance. It will also be important to continue to investigate the prevalence of HR, although epidemiologic studies are hindered by the fact that this phenotype is often undetected by clinical diagnostic tests.

## Clinically undetected HR

As mentioned previously, detection of HR by conventional antibiotic susceptibility tests is unreliable and varies greatly based on the method used. Resistant subpopulations in heteroresistant strains may be visible by gradient diffusion methods, such as disc diffusion and Etest [24], as colonies growing within the zone of inhibition. The gold-standard method to detect HR is population analysis profile, which consists of labor-intensive agar microdilution on a range of antibiotic concentrations. Unfortunately, this method is not feasible for clinical use, because of its duration and complexity. New diagnostics are needed that are sensitive enough to detect low-frequency resistant cells while maintaining the high reproducibility and low complexity that is standard for clinical testing methods.

The inability to detect HR by routine diagnostic testing results in strains being misclassified as susceptible (Fig 1, panels B and D). This can occur when the antibiotic-resistant subpopulation is present at a very low frequency. We studied one such colistin HR isolate of *E*. *cloacae* that was designated as colistin susceptible by both Etest and broth microdilution testing [14]. The resistant subpopulation in this strain was present at 1 in 10^5^ cells, which was nonetheless sufficient to mediate failure of colistin therapy in an in vivo infection model [14].

Demonstrating that this phenomenon is not restricted to *Enterobacter*, we recently identified two carbapenem-resistant *K*. *pneumoniae* (CRKP) isolates that similarly exhibited undetected colistin HR and led to failure of colistin therapy [19]. This is particularly concerning because infections with CRKP lead to high mortality rates [25], and undetected colistin HR may lead to inappropriate antibiotic prescription and unexplained treatment failure (Fig 1, panel E). In fact, even when a bacterial isolate is classified as susceptible to a given antibiotic, it is expected that antibiotic therapy will fail 10% of the time [26]. Therefore, the burden of such unexplained treatment failures is significant, and HR is a possible cause.

## Beyond antibacterials: HR to other drugs and anti-infectives

Although HR has been best characterized in bacteria, this phenomenon exists in other pathogens as well. Infectious species of *Candida* can display HR to the antifungals amphotericin B and fluconazole [27]. Pathogenic *Cryptococcus* species have been shown to develop HR to itraconazole while simultaneously gaining increased virulence and altered morphology [28]. Although parasitic pathogens have not been reported to display HR to antiparasitics, there is one report of a phenomenon similar to HR. In *Trypanosoma rhodesiense*, the pathogen responsible for African sleeping sickness, a subpopulation of cells exhibit resistance to human serum because of differential expression of a surface glycoprotein, facilitating disease in humans [29].

Phenotypic heterogeneity can also occur in cancer against chemotherapeutics [30]. Some cancers have been observed to harbor a small population of phenotypically resistant cells exhibiting chromatin modifications, allowing the cells to resist 500-times-greater concentrations of chemotherapeutic tyrosine kinase inhibitors [31]. Thus, HR or similar phenomena may explain the resistance of some cancers to chemotherapeutics when the majority of the tumor cells appear to respond to therapy.

Taken together, the current data on HR suggest that this phenomenon warrants much greater study. It is exhibited by both prokaryotes and eukaryotes, and evidence from animal infection models suggests that it can have a major impact on the outcome of therapy. The fact that HR is often undetected by clinical diagnostic tests highlights that it could be a significant cause of unexplained treatment failure. Future clinical studies will be critical to determine whether HR negatively affects outcomes in human patients. It is of the utmost importance that we thoroughly investigate all aspects of HR and potentially redefine diagnostic and treatment methods to account for the impact of these resistant subpopulations.
